# Importance of Considering Seasonality in Tick Activity When Assessing Spatial Expansion Potential: A Case Study on *Haemaphysalis longicornis*

**DOI:** 10.1002/ece3.71128

**Published:** 2025-04-23

**Authors:** Younjung Kim, Raphaëlle Métras

**Affiliations:** ^1^ Sorbonne Université, INSERM, Institut Pierre Louis d’Épidémiologie et de Santé Publique (IPLESP) Paris France

**Keywords:** Asian longhorned tick, ecological niche modelling, *Haemaphysalis longicornis*, invasive species, seasonality, spatial analysis, tick vector

## Abstract

*Haemaphysalis longicornis*, a tick vector of the severe fever with thrombocytopenia syndrome (SFTS) virus, has long been established in parts of East Asia and Oceania but is now rapidly expanding in the eastern US, raising significant concerns about its further domestic and international spread. In this study, we mapped the spatial expansion risk of *H. longicornis* in North America and Europe by training a habitat suitability model with its occurrence data from East Asia and Oceania. Our model incorporated ecologically relevant predictors for tick survival, which have been neglected in previous habitat suitability modeling for this species. Specifically, we employed temperature, relative humidity, saturation deficit, precipitation, and vegetation level as predictors, with the first three distinguishing between tick questing and non‐questing seasons to account for their varying impacts on tick survival during periods of activity and dormancy. The best‐fitting model included seasonal saturation deficit and temperature as predictors along with vegetation, with the threshold values of saturation deficit and temperature for suitability closely aligning with existing literature. Spatial projections based on the best‐fitting model most closely delineated the current boundaries of *H. longicornis* occurrences in the eastern US. In contrast, for the western US, where no *H. longicornis* has been detected yet, the model predicted only a few isolated coastal areas as suitable. This finding contrasts with earlier studies, which projected extensive suitable areas along the coast and extending inland. In Europe, the model also identified limited suitability, mainly confined to coastal areas of southern Europe. In conclusion, by incorporating meteorological predictors that account for seasonal tick activity, our findings reshape the understanding of *H. longicornis* spatial expansion risk in western North America and Europe, underscoring the need for refined and targeted surveillance strategies.

## Graphical abstract

In this study, we highlight the importance of incorporating ecologically relevant predictors when modelling habitat suitability for species with distinct seasonality, using *Haemaphysalis longicornis* ticks as a case study and drawing stark comparisons to earlier studies.

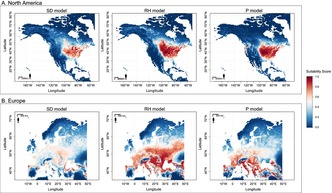

## 1 Introduction

*Haemaphysalis longicornis* , a tick from the Ixodidae family, is responsible for transmitting the severe fever with thrombocytopenia syndrome (SFTS) virus to humans and other animals, with human cases increasingly being notified in East Asia (Cui et al. 2024). *H longicornis* has an extensive native habitat range across East Asia and has established populations in Oceania for several decades. Outside these regions, *H. longicornis* was first detected in large numbers and across all life stages in the eastern US in 2017 (Rainey et al. 2018). However, an evaluation of historical samples indicates its presence there as early as 2010 (Thompson et al. 2022). As of July 2024, its identified habitat has rapidly expanded to 21 eastern US states (US Department of Agriculture's Animal and Plant Health Inspection Service (APHIS) 2024), raising significant concerns about how much farther this medically important vector could spread in North America and beyond.

To date, four studies have assessed *H. longicornis* habitat suitability, all conducted within several years after it began to be collected in large numbers in North America (Namgyal et al. 2020; Raghavan et al. 2019; Rochlin 2018; Zhao et al. 2020). These earlier studies fitted species distribution models to data on *H. longicornis* occurrences in East Asia and Oceania—where *H. longicornis* has established—using time‐independent meteorological predictors such as long‐term averages of temperature, precipitation (Namgyal et al. 2020; Rochlin 2018; Zhao et al. 2020) or specific humidity (Raghavan et al. 2019), along with other time‐independent predictors such as land cover (Zhao et al. 2020) and ecological zones (Namgyal et al. 2020; Rochlin 2018). Then, based on model results, *H. longicornis* habitat suitability was projected on a global scale in one study (Zhao et al. 2020) and for North America in the other three studies (Namgyal et al. 2020; Raghavan et al. 2019; Rochlin 2018). All studies identified extensive regions of eastern North America, and coastal and inland regions of western North America (Namgyal et al. 2020; Raghavan et al. 2019; Rochlin 2018; Zhao et al. 2020) as suitable for *H. longicornis* . However, while *H. longicornis* has been rapidly expanding in the eastern US since its first detection in 2017 (US Department of Agriculture's Animal and Plant Health Inspection Service (APHIS) 2024), it has not yet been detected in the western part of the country, contrary to the consistent predictions made by the earlier studies (Namgyal et al. 2020; Raghavan et al. 2019; Rochlin 2018; Zhao et al. 2020).

In this study, we hypothesised that the discrepancy between the predictions of the earlier studies and the distribution of *H. longicornis* in the western US until 2024 may stem from the fact that these models did not explicitly account for the species' seasonal activities (i.e., phenology), despite this being a critical factor for tick survival. To address this potential limitation, we modelled *H. longicornis* habitat suitability by incorporating meteorological conditions that reflect the species' phenology, such as periods of activity and dormancy, and reassessed its spatial expansion risk for two Northern Hemisphere continents: North America, where it is rapidly expanding, and Europe, where it has not yet been detected.

## 2 Material and Methods

### 2.1 *Haemaphysalis longicornis* Occurrence Data

We used *H. longicornis* occurrence data from two of the earlier studies that modelled *H. longicornis* habitat suitability (Rochlin 2018; Zhao et al. 2020). These geocoordinate data were considered sufficient to represent the geographical extents of *H. longicornis* habitats in native regions, such as in East Asia and Oceania, thereby making them suitable for model fitting (Elith and Leathwick 2009). We did not include data from the other two earlier studies because, in one study, the occurrence data were sourced from the same studies we used (Namgyal et al. 2020) and in the other, the occurrence data were not publicly available (Raghavan et al. 2019).

To reduce the risk of spatial autocorrelation, we filtered *H. longicornis* occurrence data with the *thin* function from the *spThin* package (Aiello‐Lammens et al. 2015) in R 4.3.2 (R Core Team 2023). We applied a 30 km distance threshold to ensure that no two occurrence points fall within the same pixel representing meteorological variables (each pixel being approximately 28 × 28 km). To account for the randomness resulting from this filtering procedure and to robustly compare our habitat suitability models through model fitting, we generated 100 independently filtered datasets.

### 2.2 Climate Variables

We first selected the following meteorological variables: (i) monthly averaged soil temperature, (ii) monthly averaged relative humidity, (iii) monthly averaged saturation deficit, (iv) monthly averaged vegetation, and (v) monthly total precipitation. All meteorological data, except for saturation deficit, were obtained directly from the Copernicus Climate Change Service (Hersbach et al. 2023; Muñoz Sabater 2019). The relative humidity data were obtained from ERA5 monthly averaged data on pressure levels (Hersbach et al. 2023), while the temperature (*soil temperature level 1*), vegetation (*Leaf area index, low vegetation*), and total precipitation data were sourced from ERA5‐Land monthly averaged data (Muñoz Sabater 2019). To ensure consistency across meteorological variables, the ERA5‐Land datasets were resampled to match the spatial resolution of the relative humidity data using the bilinear interpolation method with the *resample* function of the *raster* package (Hijmans 2023) in R 4.3.2 (R Core Team 2023). Saturation deficit represents the drying power of the air (Randolph 2004), and we computed monthly averaged saturation deficit using monthly temperature and relative humidity using the formula provided by Randolph and Storey (1999): $\mathrm{SD}=\left(1-\frac{\text{relative humidity}}{100}\right)\times 4.9463e^{0.0621\times \text{temperature}}$ For each variable, its monthly values were then averaged from 2015 to 2023 by calendar month.

Temperature and humidity levels are critical for *H. longicornis* survival through the modulation of water balance in the body and development, and these two conditions together determine saturation deficit representing the drying power of air (Schappach et al. 2020). Low saturation deficit values are associated with favourable environments (Schappach et al. 2020). Vegetation level was included as it reflects the underlying ecosystem suitable for ticks, providing places for hiding and questing (i.e., a phase during which ticks seek a host for a blood meal) as well as influencing host presence. Precipitation level was included for its influence on tick survival, affecting humidity levels, and potentially causing mortality during heavy rainfall (Weiler et al. 2017). Precipitation is also included in our study primarily to develop a model consistent with the earlier studies and to compare its projections with those of our other models (see the P model in “ *H. longicornis* habitat suitability model” below).

Importantly, we distinguished temperature, relative humidity, and saturation deficit by the questing and non‐questing seasons and assigned the maximum or minimum monthly values to each pixel. More specifically, for temperature, we used the maximum value from the questing season and the minimum from the non‐questing season as predictors, acknowledging that temperature may impact tick survival differently between these periods. Then, for saturation deficit, for the questing season, we used its maximum value, assuming that it reflects the upper thresholds for tick survival. For relative humidity, we used its minimum values instead, considering its inverse relationship with saturation deficit. For precipitation and vegetation levels, we used the mean of the monthly averages without differentiation by season, assuming that their influence remains constant throughout the year. This approach allows us to uniquely account for the differential impacts of those meteorological variables on tick survival and activity, making it a key methodological refinement compared to previous studies (Namgyal et al. 2020; Raghavan et al. 2019; Rochlin 2018; Zhao et al. 2020). The questing seasons were determined based on the literature for the regions where *H. longicornis* has long been established—April to October for East Asia (Johnson et al. 2017) and February to August for Oceania (Heath 2016). When projecting *H. longicornis* habitat suitability for North America and Europe, the same periods as East Asia were assumed, given that all these regions are in the Northern Hemisphere.

Finally, a pairwise Pearson's correlation analysis was performed for all considered meteorological variables. Among the pairs that were highly correlated (Pearson's *r* > 0.8), one variable was removed based on its relative ecological significance for tick survival.

### 2.3 *Haemaphysalis longicornis* Habitat Suitability Model

We modelled *H. longicornis* habitat suitability using Maxent software v3.4.3 (Phillips and Dudík 2008). This approach was also used in the earlier studies, ensuring our predictions to be comparable to theirs (Namgyal et al. 2020; Raghavan et al. 2019; Rochlin 2018; Zhao et al. 2020). We trained the model on the *H. longicornis* occurrence data in East Asia and Oceania—regions where this tick is established (Rochlin 2018; Zhao et al. 2020)—using three distinct sets of meteorological predictors.

As baseline meteorological variables, we included the maximum temperature during the questing season, the minimum temperature during the non‐questing season and the mean vegetation (not differentiated by season), given their importance for *H. longicornis* survival (Schappach et al. 2020). Building on these, we tested three models: a saturation deficit model (SD model), a relative humidity model (RH model), and a precipitation model (P model).

In the SD and RH models, respectively, saturation deficit and relative humidity values, both during the questing season, were used to capture their significance in that season. During the questing season, maximum temperature and saturation deficit values were highly correlated (Pearson's *r* = 0.85) (Table S1). However, considering that each variable can influence different aspects of tick survival, both were retained in the SD model. In the P model, precipitation was not separated by questing versus non‐questing seasons so as to align with the earlier studies and therefore allow the comparison of P model projections with both our SD and RH models, as well as with the earlier studies.

Each model was fitted to 100 independently filtered datasets. In each model fitting, 25% of the occurrences were assigned as test points, while the remaining occurrences were used as training points. Linear, quadratic, product and hinge features were allowed to allow biotic interactions, which could potentially affect tick survival. The models were allowed to iterate up to 10,000 times to ensure convergence, and the robustness of the model results was also confirmed by cross‐validation through 25 runs.

The final model comparisons were based on the mean corrected Akaike's information criterion (AICc) value across these 100 filtered datasets, with the model yielding the lowest mean AICc selected as the best‐fitting model. We then chose the filtered dataset that produced the lowest AICc for projections with the best‐fitting model. First, we projected *H. longicornis* habitat suitability for East Asia and Oceania regions where the *H. longicornis* occurrence data originated, and subsequently for two Northern Hemisphere continents: North America, where *H. longicornis* is currently invading, and Europe, where the tick has not been reported yet.

## 3 Results

After applying a 30 km distance threshold, the filtered datasets included a median of 323 occurrences (range: 320–325), compared to 550 occurrences in the original dataset from East Asia and Oceania. Both the SD and RH models yielded significantly lower mean AICc values than the P model, with the SD model producing the lowest mean AICc value and therefore being selected as the best‐fitting model (AICc: 445.8, 457.8, and 477.8 for the SD, RH, and P models, respectively).

**FIGURE 1:**
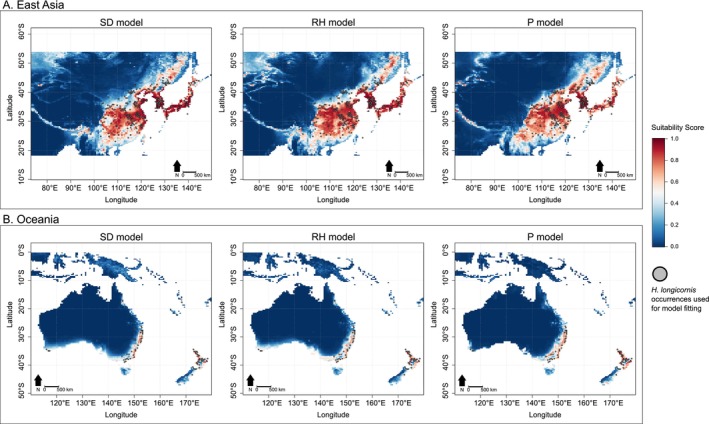
Predicted habitat suitability for *Haemaphysalis longicornis* in (A) East Asia and (B) Oceania by the saturation deficit (SD), relative humidity (RH), and precipitation (P) models. The grey circles represent the locations of *H. longicornis* to which the three models were fitted using Maxent v.3.4.3 (Phillips and Dudík 2008).

**FIGURE 2:**
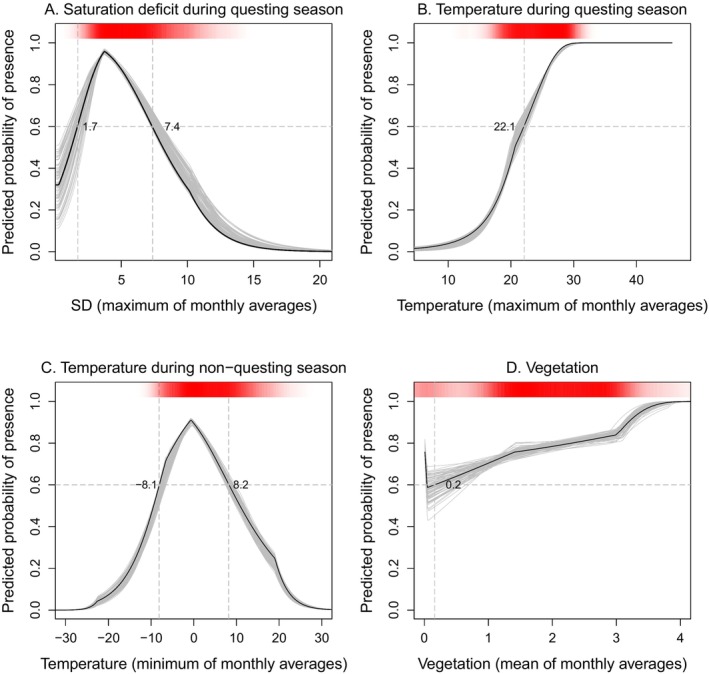
Relationship between predictors and the probability of presence for the best‐fitting model (SD Model). (A) Saturation deficit during the questing season, (B) temperature during the questing season, (C) temperature during the no‐nquesting season, and (D) vegegation. The lines show changes in the predictive probability of *Haemaphysalis longicornis* presence as a function of each predictor, accounting for the effects of other predictors. The black line is from the filtered dataset that produced the lowest corrected Akaike's information criterion value, and the grey lines are from the remaining 99 filtered datasets. The dashed horizontal and vertical lines indicate the point where the predictive probability reaches 0.6, above which regions are generally considered highly suitable. The reddish shades represent *H. longicornis* occurrence data, with density corresponding to the frequency of occurrences at given predictor values.

Our projections for East Asia and Oceania, where the *H. longicornis* occurrence data originated, demonstrated strong predictive power across all models (Area Under the Curve [AUC]: 0.915, 0.912, and 0.916 for SD, RH, and P model, respectively, when fitted to the dataset that produced the lowest AICc by the SD model). The SD model projected the most restricted highly suitable regions in both East Asia and Oceania. The differences between these projections were particularly pronounced in southern China (Figure 1A). In the SD model, saturation deficit during the questing season (mean: 35.1%, range: 32.2%–37.1%) was identified as the most important predictor, along with temperature during the questing season (31.0%, 29.9%–32.6%) and temperature during the non‐questing season (32.6%, range: 31.0%–35.1%) (Table S2). Notably, the SD model suggested that during the questing season, areas with a saturation deficit below 7.4 were suitable when suitability was defined as a predicted probability of presence > 0.6 following the classification by Namgyal et al. (2020) (Figure 2A). The SD model also indicated that areas with higher temperatures were more suitable during the questing season (Figure 2B), while temperatures below −8.1°C were considered unsuitable during the non‐questing season (Figure 2C) (see Figures S1 and S2 for the RH and P model results).

**FIGURE 3:**
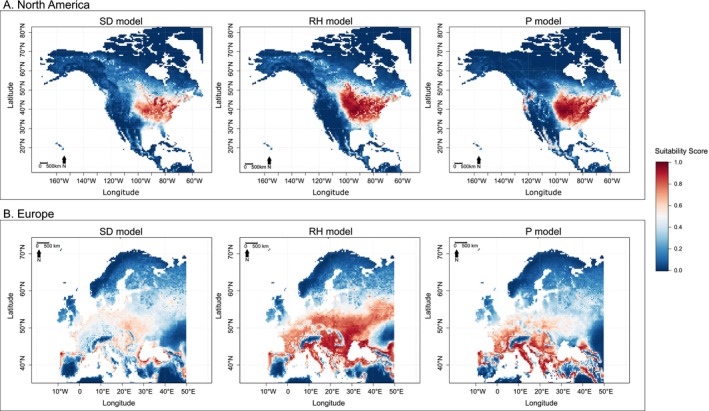
Predicted habitat suitability for *Haemaphysalis longicornis* in (A) North America and (B) Europe by the saturation deficit (SD), relative humidity (RH), and precipitation (P) models. The occurrences in the eastern US are not displayed here due to copyright restrictions but are available here: https://www.aphis.usDa.gov/livestock‐poultry‐disease/cattle/ticks/asian‐longhorned.

**FIGURE 4:**
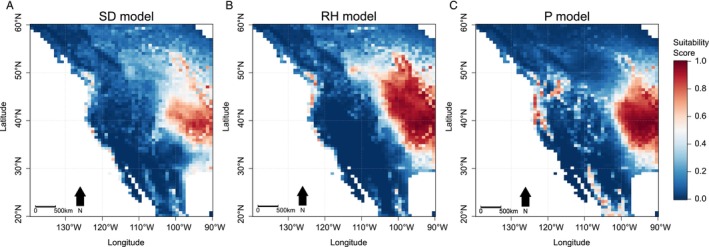
Predicted habitat suitability for *Haemaphysalis longicornis* in western North America by the (A) saturation deficit (SD), (B) relative humidity (RH), and (C) precipitation (P) models. The projection by the P model is similar to those of the four studies that assessed *H. longicornis* habitat suitability (Namgyal et al. 2020; Raghavan et al. 2019; Rochlin 2018; Zhao et al. 2020).

In North America (Figures 3A and 4) and Europe (Figure 3B), the SD, RH, and P models projected markedly different pictures of *H. longicornis* habitat suitability. The SD model, offering the best fit, also exhibited the most restrictive spatial projections. The largest difference is observed in the projections for western North America, where *H. longicornis* has not yet been detected (Figures 3A and 4). Whilst earlier studies (Namgyal et al. 2020; Raghavan et al. 2019; Rochlin 2018; Zhao et al. 2020)—and their counterpart P model—projected large suitable areas along the coast and extending inland, the RH and SD models identified only a few isolated coastal areas as suitable in this region (Figures 3A and 4). In contrast, in eastern North America, all models agreed on high suitability for areas with reported *H. longicornis* occurrences, but the spatial extent of suitable areas was most restrictive with the SD model compared to the RH and P models (Figure 3). Importantly, the highly suitable regions identified by the SD model successfully captured *H. longicornis* occurrences in the eastern US until 2024, as documented in the government's situation report (US Department of Agriculture's Animal and Plant Health Inspection Service (APHIS) 2024). These occurrences are not displayed here due to copyright restrictions but are available here: https://www.aphis.usda.gov/livestock‐poultry‐disease/cattle/ticks/asian‐longhorned. Furthermore, its projections delineated the southernmost and northernmost boundaries of these occurrences, suggesting a potential gradient in the species' expansion within the eastern U.S. (Figure 3A) (US Department of Agriculture's Animal and Plant Health Inspection Service (APHIS) 2024).

For Europe, the models also diverged sharply, with the SD model showing a dramatic reduction in suitability compared to the RH and P models (Figure 3B). While the RH and P models identified broader suitable regions, extending to southeast England, the SD model predicted suitability only in some coastal areas of southern Europe (Figure 3B).

## 4 Discussion

By accounting for ticks' seasonal activity, our findings indicate that the risk of *H. longicornis* invasion in the western US and Europe is likely lower than previously predicted, while agreeing with its spatial expansion in the eastern US observed until 2024.

This difference compared to the projections from earlier studies may be attributed to the fact that the survival—and thus habitat suitability—of *H. longicornis* is strongly influenced by the interaction between its seasonal activities and climate patterns (Fielden and Lighton 1996; Perret et al. 2003; Randolph 2004). In these earlier studies, the way meteorological predictors, such as precipitation and specific humidity, were considered may not have adequately captured these important features (Namgyal et al. 2020; Raghavan et al. 2019; Rochlin 2018; Zhao et al. 2020).

**FIGURE 5:**
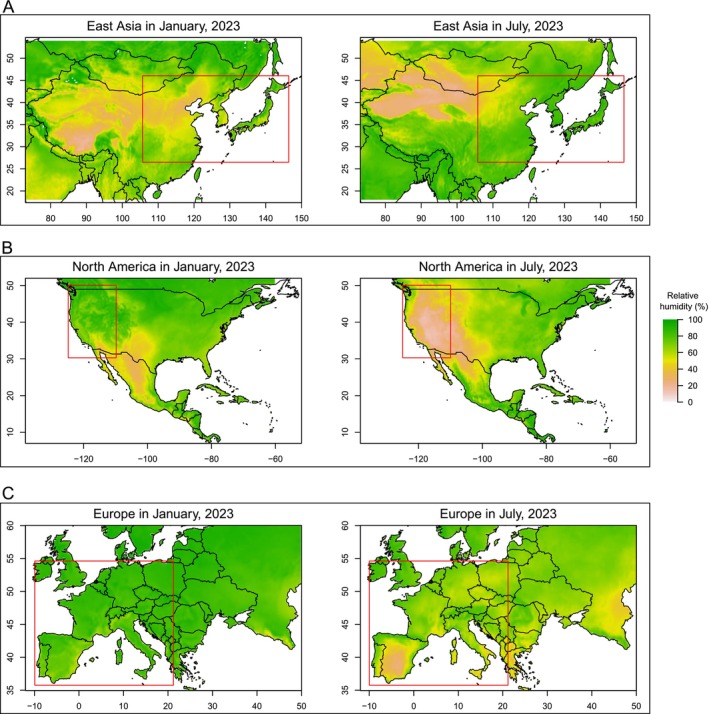
Comparison of relative humidity between January and July 2023, in (A) East Asia, (B) North America and (C) Europe across different seasons in 2023. (A) In eastern China, Korea, and Japan, where *Haemaphysalis longicornis* is widespread, the level of relative humidity is much higher in July (during the questing season) than in January (during the non‐questing season). This trend is obviously reversed in western North America (B) and Europe (C). The red rectangles highlight the regions with significant differences between January and July 2023. The relative humidity data is obtained from the Copernicus Climate Change Service ERA5 monthly averaged data on pressure levels (Hersbach et al. 2023).

Firstly, high precipitation used previously may not necessarily reflect high moisture levels suitable for *H. longicornis* establishment and survival, as regions with intense but infrequent rainfall in summer might still have low humidity. Combined with elevated temperatures, such conditions could still result in a high drying power of the air, leaving ticks prone to desiccation. Secondly, the precipitation and specific humidity metrics previously used—such as annual totals or amounts during the driest or wettest periods—do not adequately capture the meteorological conditions affecting ticks' ability to maintain water balance (Ponti and Sannolo 2023). Beyond the fact that annual totals obscure seasonal effects, without temperature information, the metrics from the driest or wettest seasons are insufficient to capture the climate conditions suitable for tick questing, as these seasons vary by region of the globe (Hersbach et al. 2023). For example, in East Asia, the wettest season does align with the questing season of *H. longicornis* in summer. However, in western North America and Europe, the wettest season occurs during winter, which is not the major questing season (see Figure 5 for this stark comparison).

It is worth noting that the SD model, which incorporated saturation deficit, temperature, and vegetation as predictors, provided the most restrictive spatial predictions for North America and Europe compared to those of the earlier studies and the corresponding P model. Notably, the predictions for North America closely delineated the boundaries of *H. longicornis* occurrences observed until 2024 (US Department of Agriculture's Animal and Plant Health Inspection Service (APHIS) 2024) (see https://www.aphis.usda.gov/livestock‐poultry‐disease/cattle/ticks/asian‐longhorned). This may suggest that saturation deficit serves as a conservative and effective predictor for *H. longicornis* habitat suitability.

Additionally, the SD model identified saturation deficit during the questing season as the most important predictor, along with temperature. This finding is in line with the current knowledge of *H. longicornis* biology, most importantly because ticks would face a high risk of dehydration—and potentially death—if they attempted questing or developing under conditions of high saturation deficit (Kahl and Gray 2023; Schappach et al. 2020). Conversely, even at elevated temperatures, high humidity reduces the risk of dehydration, as demonstrated in laboratory experiments showing increased developmental rates at higher temperatures under near‐saturated humidity for other Ixodidae ticks (Ogden et al. 2004; Randolph et al. 2002). This likely explains why saturation deficit, rather than temperature, during the questing season contributed most significantly to the model's fit. Supporting this, our estimate of a saturation deficit below 7.4, indicating suitable conditions, closely aligns with the upper threshold values reported in the literature for *H. longicornis* eggs and larvae (Heath 1979, 1981; Schappach et al. 2020).

Finally, for the non‐questing period, temperature was identified as the most significant predictor, along with saturation deficit and temperature during the questing season. This may suggest that, when the risk of dehydration is low due to relatively low temperatures, the lower temperature threshold becomes a critical factor for *H. longicornis* survival (Yu et al. 2014). Further supporting our findings, the lower temperature threshold of −8.1°C was consistent with the temperature at which survival rates began to decrease in laboratory experiments (Yu et al. 2014).

There are several areas for improvement. First, our analysis assumed the same level of environmental heterogeneity across the study region. To minimise potential spatial autocorrelation, we used a 30 km distance threshold in accordance with a pixel size, which resulted in the removal of a significant proportion of occurrences from the original data. However, this approach might have led to the loss of some occurrence information in highly heterogeneous regions, while autocorrelation could persist in overly homogeneous regions. Second, while survey time was not available for the occurrence data we used, there is a need to fit a habitat suitability model to more recent *H. longicornis* occurrences in East Asia and Oceania to better understand the species' current adaptation, if present, to changing climate in its native habitat. Lastly, we fitted the model to the *H. longicornis* occurrence data in East Asia and Oceania, considering that populations in these regions are at equilibrium with their environment—a key assumption of habitat suitability modelling (Elith and Leathwick 2009). In contrast, *H. longicornis* populations in the US are considered to be in a phase of rapid expansion and therefore not yet at equilibrium. However, future research could include regions in the eastern US when *H. longicornis* populations can be considered stable at equilibrium, as this would likely improve predictions for the US by incorporating meteorological and ecological data that better reflect regional conditions.

Our findings underscore the importance of considering a species' life cycle, particularly its seasonal activities, when incorporating meteorological predictors into habitat suitability modelling (Ponti and Sannolo 2023). While the availability of extensive meteorological data makes habitat suitability modelling more accessible, these data must reflect the species' life cycle and thereby phenology. This consideration is especially crucial when the results of habitat suitability modelling are intended to inform surveillance systems and control measures. Our results support more focused surveillance efforts, for example, in the eastern US. Surveillance efforts may need a different focus in the western US. It would be reasonable to assume that, while *H. longicornis* has been established and further expanding in the eastern states, this species may have also been introduced but failed to establish in the western states. Given this, the absence of identified populations in the western states is likely primarily driven by limited habitat suitability rather than insufficient time for establishment. Therefore, in this region, continuous monitoring of climate change and its impact on habitat suitability should be considered.

In conclusion, by accounting for ticks' seasonal activity, our findings indicate that the risk of *H. longicornis* invasion in the western US and Europe is likely lower than predicted by previous models. Our findings also underscore the critical importance of selecting meteorological predictors that are ecologically relevant to the phenology of the species in question when modeling its habitat suitability, to improve surveillance and risk management strategies for tick vectors and the diseases they spread.

## Author Contributions

**Younjung Kim:** conceptualization (lead), formal analysis (lead), investigation (lead), methodology (lead), visualization (lead), writing – original draft (lead), writing – review and editing (equal). **Raphaëlle Métras:** conceptualization (supporting), methodology (supporting), visualization (supporting), writing – review and editing (equal).

## Conflicts of Interest

The authors declare no conflicts of interest.

## Supporting information


**Data S1.** Supporting Information.

## Data Availability

All meteorological data were obtained directly from the Copernicus Climate Change Service, which is publicly available (Hersbach et al. 2023; Muñoz Sabater 2019). *Haemaphysalis longicornis* occurrence data were collated from Rochlin (2018) and Zhao et al. (2020).
