# Supplementary material for: Importance of Considering Seasonality in Tick Activity When Assessing Spatial Expansion Potential: A Case Study on Haemaphysalis longicornis
Source: Ecol Evol. 2025 Apr 23;15(4):e71128. doi: 10.1002/ece3.71128 (PMC12015634; doi:10.1002/ece3.71128)
Supplement: Supplementary file 1 — Data S1. Supporting Information. [file ECE3-15-e71128-s001.docx]

**Supplementary Information for**

**Importance of incorporating seasonal variations in tick activity in assessing spatial expansion potential: A case study of *Haemaphysalis longicornis***

Younjung Kim^1^, Raphaëlle Métras^1^

^1^ Sorbonne Université, INSERM, Institut Pierre Louis d’Épidémiologie et de Santé Publique (IPLESP), UMRS 1136, Paris, France

Dr Younjung Kim ([younjung.kim@inserm.fr](mailto:younjung.kim@inserm.fr))

**Supplementary Figures**


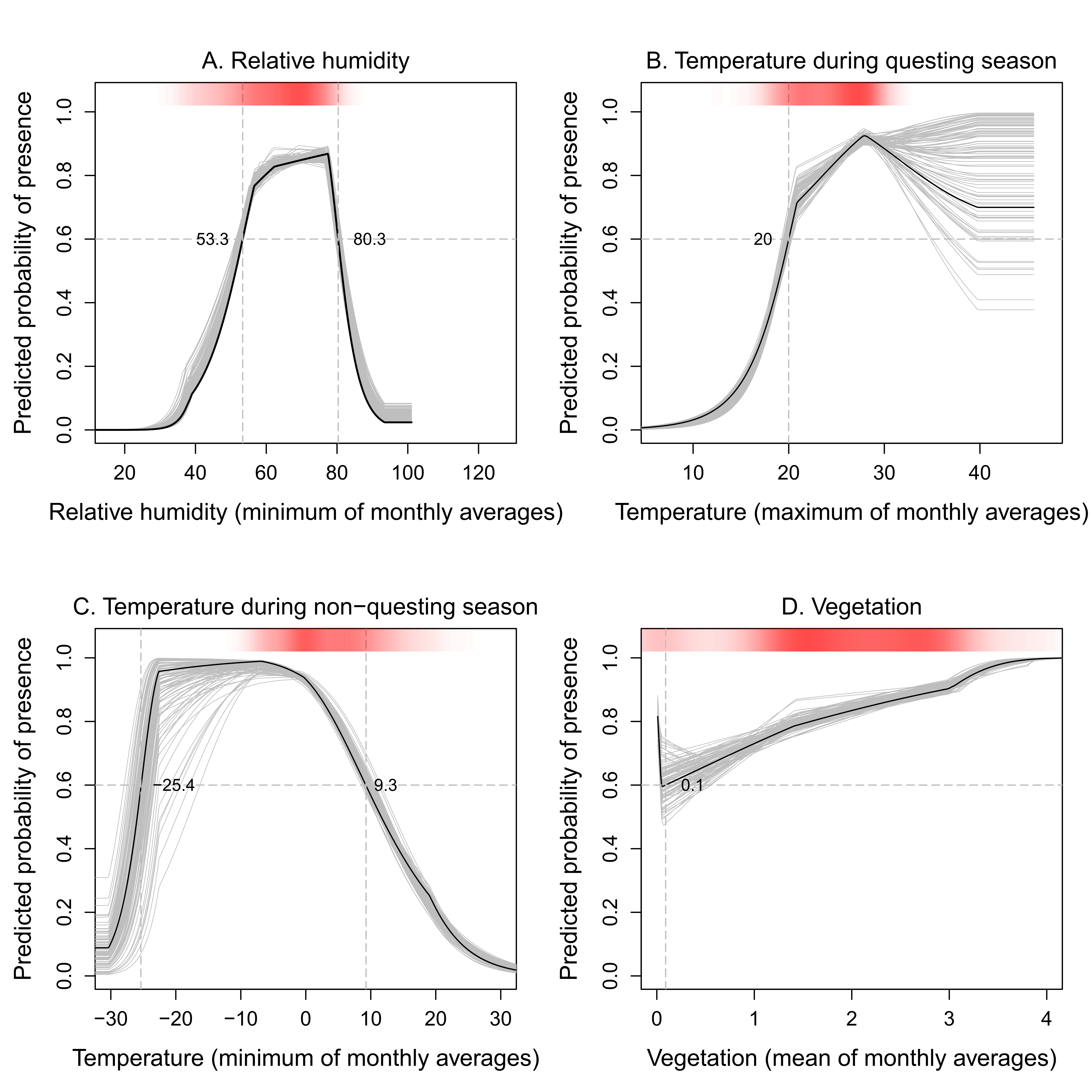


**Figure S1.** Results of the RH model. (A) displays the relative contributions of individual predictors to the relative humidity (RH) model (read permutation importance score). The solid lines illustrate changes in the predictive probability of Haemaphysalis longicornis presence as a function of each predictor, accounting for the effects of other predictors. The black solid line is from the filtered dataset that produced the lowest corrected Akaike’s information criterion value, with the dashed lines indicating the point where the predictive probability reaches 0.6, above which regions are generally considered highly suitable. The grey solid lines are from the remaining 99 filtered datasets. The reddish shades represent H. longicornis occurrence data, with density corresponding to the frequency of occurrences at given predictor values.


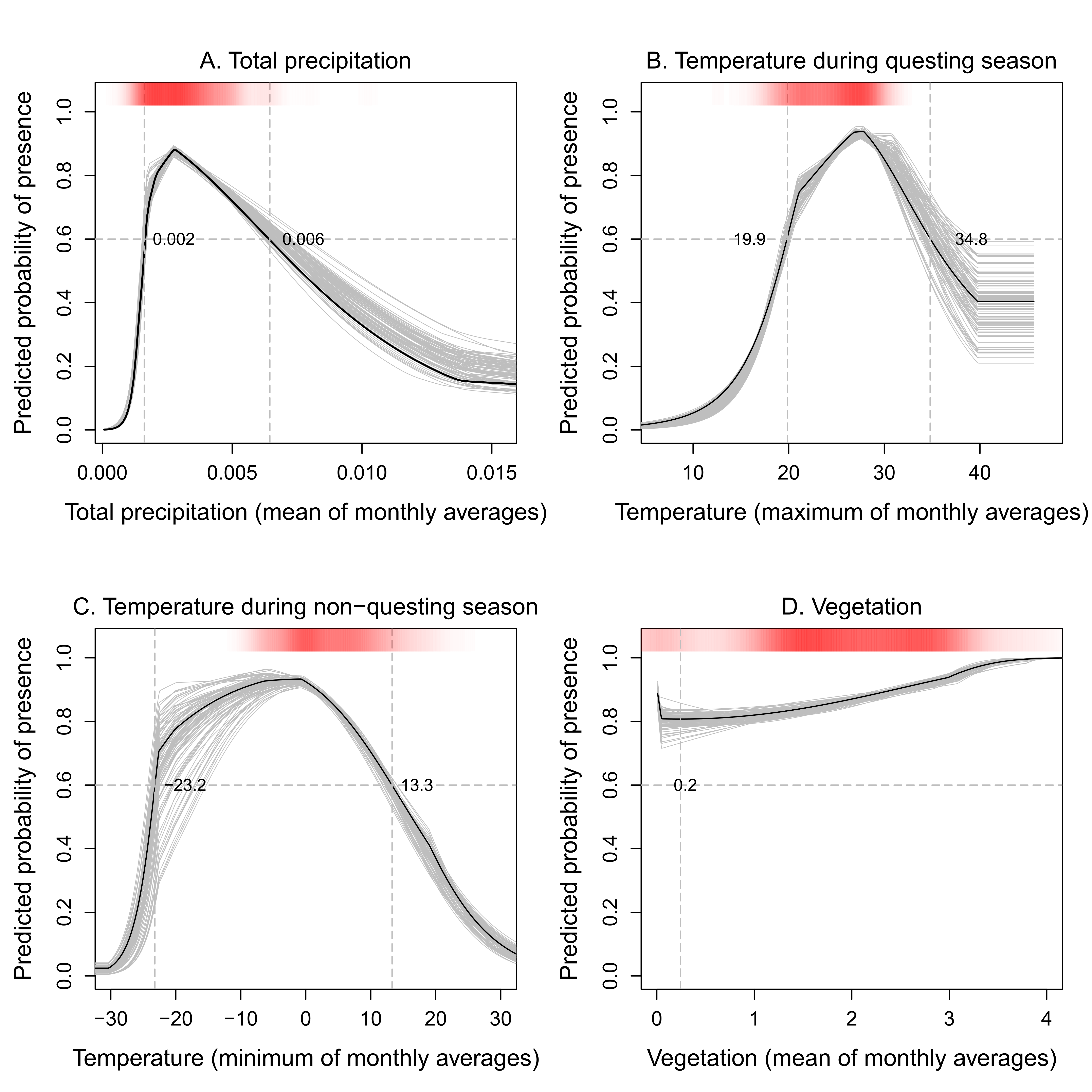


**Figure S2.** Results of the P model. The solid lines illustrate changes in the predictive probability of Haemaphysalis longicornis presence as a function of each predictor, accounting for the effects of other predictors. The black solid line is from the filtered dataset that produced the lowest corrected Akaike’s information criterion value, with the dashed lines indicating the point where the predictive probability reaches 0.6, above which regions are generally considered highly suitable. The grey solid lines are from the remaining 99 filtered datasets. The reddish shades represent H. longicornis occurrence data, with density corresponding to the frequency of occurrences at given predictor values.

| **Table S1.** Pairwise Pearson correlation coefficient between environmental variables used for *H. longicornis* habitat suitability models | | | | | | |
| --- | --- | --- | --- | --- | --- | --- |
|  | Mean vegetation | Mean total precipitation | Maximum soil temperature  (questing season) | Minimum soil temperature  (non-questing season) | Minimum relative humidity  (questing season) | Maximum saturation deficit (questing season) |
| Mean vegetation |  | 0.40 | -0.02 | 0.18 | 0.43 | -0.25 |
| Mean total precipitation | 0.40 |  | -0.10 | 0.29 | 0.71 | -0.38 |
| Maximum soil temperature (questing season) | -0.02 | -0.10 |  | 0.65 | -0.44 | 0.85 |
| Minimum soil temperature  (non-questing season) | 0.18 | 0.29 | 0.65 |  | 0.10 | 0.46 |
| Minimum relative humidity (questing season) | 0.43 | 0.71 | -0.44 | 0.10 |  | -0.72 |
| Maximum saturation deficit (questing season) | -0.25 | -0.38 | 0.85 ^a^ | 0.46 | -0.72 |  |
| a Maximum soil temperature during the question season was maintained in the SD model given its ecological significance for *H. longicornis survival*. | | | | | | |

| **Table S2.** Relative contribution of the meteorological variables in the SD, RH, and P models | |
| --- | --- |
|  | Permutation importance (mean, [range]) |
| SD model |  |
| Mean vegetation | 1.3 [0.7-2.0] |
| Maximum soil temperature (questing season) | 31.0 [29.9-32.6] |
| Minimum soil temperature (non-questing season) | 32.6 [31.0-35.1] |
| Maximum saturation deficit (questing season) | 35.1 [32.2-37.1] |
|  |  |
| RH model |  |
| Mean vegetation | 2.0 [1.0-3.8] |
| Maximum soil temperature (questing season) | 30.0 [28.4-31.7] |
| Minimum soil temperature (non-questing season) | 30.3 [28.0-33.2] |
| Minimum relative humidity (questing season) | 37.8 [32.9-41.4] |
|  |  |
| P model |  |
| Mean vegetation | 0.9 [0.5-1.4] |
| Maximum soil temperature (questing season) | 30.0 [28.7-31.9] |
| Minimum soil temperature (non-questing season) | 30.8 [29.1-32.7] |
| Mean total precipitation | 38.2 [34.8-40.3] |
| The mean and range are obtained from model fitting to 100 filtered datasets. | |
